# Efficacy and potential mechanism of atherosclerosis prevention by the active components of leech based on network pharmacology combined with animal experiments

**DOI:** 10.1016/j.heliyon.2024.e27461

**Published:** 2024-03-08

**Authors:** Qing Lv, Mengyi Li, Ziyun Wen, Qianqian Han, Liang Wei, Jisheng Chen, Yunyun Pan

**Affiliations:** a Clinical Pharmacy Center, Nanfang Hospital, Southern Medical University, Guangzhou, 510515, China; b The First Affiliated Hospital of Guangdong Pharmaceutical University, Guangzhou, 510080, China

**Keywords:** Network pharmacology, Leech, Atherosclerosis, Molecular docking, Plaque stability

## Abstract

**Introduction:**

Leeches are flesh-eating and bloodsucking parasitic worms. They are being used as a traditional Chinese medicine for centuries in activating blood and dissolving statis, dreging the meridims and tick. Hirudin, an active peptide product present in leech, has blood anticoagulant property and can assist in the treatment of thrombosis and diseases related to blood circulation. The efficacy and potential mechanism of action of leeches in such diseases should be further explored.

**Materials and methods:**

First, network pharmacology was used to screen the predicted potential targets of the active constituents of leech and AS. The common targets of the active constituents of leech and AS were obtained using Venn diagram. Further, the drug-active-constituent–target network diagram, protein–protein interaction, and GO and KEGG pathway enrichment analyses were used to construct the active-constituent–AS target–pathway network diagram. Subsequently, the protein–drug molecule docking model was drawn. Finally, the results of network pharmacology were validated using a mouse model of AS.

**Results:**

In total, 34 active constituents of leech and 1172 AS-related gene targets were selected, took the drug action targets and potential disease targets to get the common targets, and took the top 10 of degree value as the main active constituents for the treatment of atherosclerosis. There were 89 common targets and 12 core targets. The main targets included MAPK, EGFR, PIK3CB, etc. Potential regulatory pathways included cancer pathways, EGFR tyrosine kinase inhibitor resistance, Rap1 signaling pathway, PPAR signaling pathway, PI3K-Akt signaling pathway, C-type lectin receptor signaling pathway, and AGE-RAGE signaling pathway in diabetic complications. Animal experiments using mouse model of AS confirmed that AS plaques were smaller after treatment with leeches. SRC level was measured using western blotting. Expression of SRC in myocardial tissue was remarkably lower in the mice treated with leech than in the mice from model group fed on high-fat chow.

**Conclusions:**

To the best of our knowledge, this is the first study to explore the mechanism of action of the active components of leech in AS prevention. The active components of leeches play a coordinated role in preventing AS through multicomponent, multitarget, and multichannel mechanism of action related to inflammatory response, oxidative stress, and lipid metabolism. This study provided a reference for subsequent cellular and animal experiments.

## Graphical abstract

**Figure:**
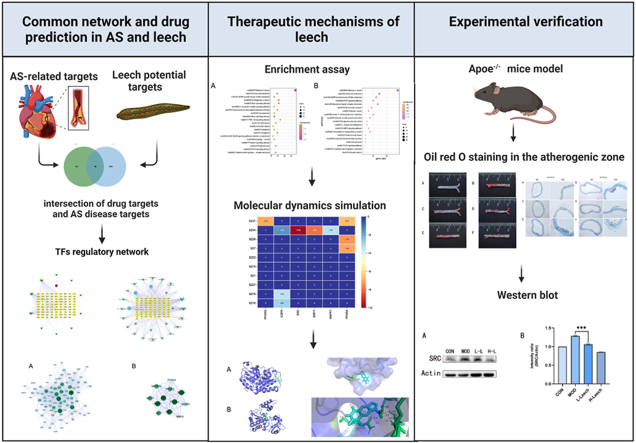

## 1 Introduction

Leeches are flesh-eating and bloodsucking worms and are used as traditional Chinese medicine. They are harsh and fierce, with unique effects such as activating blood and dissolving statis, dreging the meridims and tick, etc. Leeches play a unique role in the treatment of atherosclerosis (AS), and their action cannot be replaced by other drugs [1]. According to the theory of “stasis and lipid toxicity,” stasis toxicity is the important mechanism of action for the formation of vulnerable plaques in AS, and activating blood, resolving stasis and toxicity is the basic treatment for stabilizing vulnerable plaques [2]. Zhang explored the medication rules of traditional Chinese medicine in the treatment of AS. Among the 109 selected literatures, almost 20.9% of the references were about drugs for activating blood and dissolving statis. Zhang et al. confirmed that activating blood and dissolving statis was the basic method for the treatment of AS [3]. Leech is a representative drug in activating blood and dissolving statis, dreging the meridims and tick [1,4,5].

Leech was recommended as a reliable and effective procedure for several painful and inflammatory disorders [6,7]. Previous research has shown that leeches have antimicrobial effectsAnticancer effects, effects against cell degradation, application for skin flaps, wound healing improvement, usage in reconstructive surgeries, acute venous congestion recovery, and shielding effects against cerebral ischemiareperfusion injury [[8], [9], [10], [11], [12]].

Recent studies have reported that leeches exhibit multicomponent and multitarget interventions in AS by inhibiting the proliferation of vascular smooth muscle cells, regulating lipid metabolism, protecting vascular endothelium, and inhibiting inflammation [13].

AS remains a major life-threatening disease globally. Several individuals succumb to the acute complications of AS, despite advances in prevention strategies [14]. AS involves lesions related to impaired lipid metabolism, and its progression involves various pathological factors such as vascular endothelial cells, inflammatory response, and oxidative stress [15].

In this study, we demonstrated the mechanism of action of the active components of leech in AS prevention via a concise analytical method involving network pharmacology. Furthermore, animal experiments were performed using a mouse model of AS to verify the active constituents of leech, targets, and pathways predicted by network pharmacology. This study provided a basis to analyze the active constituents and targets of leeches against AS more systematically through bioinformatics and network pharmacology analysis.

## 2 Materials and methods

### 2.1 Collection and screening of active constituents and targets of leeches

A database of leech active ingredients was established by referring to the literature on leech chemical composition [[16], [17], [18], [19], [20], [21], [22], [23], [24], [25]],the SMILES structure was obtained by searching the leech active constituent PubChem database [26] (https://pubchem.ncbi.nlm.nih.gov/).

Further, SDF files of the SMILES structure were uploaded to the Swiss ADME database [27] (http://www.swissadme.ch/) for constituent screening with the following conditions. High gastrointestinal absorption and drug-likeness of an active constituent were predicted using Lipinski, Ghose, Veber, Egan, and Muegge rules; if it met 2 items (as Yes), this leech active constituent had high oral utilization.

The potential targets of the predicted active constituents of leech were screened using Swiss Target Prediction database [27] (http://www.Swisstargetprediction.ch/). The targets with probability <10% were excluded.

### 2.2 AS target screening

Using the GeneCards database [28], with “atherosclerosis” as the keyword, targets of AS were screened. Those with a higher score value indicated that they were more closely associated with AS. Empirically, in case of several targets, the target with score greater than twice the median was set as a potential target for AS. In this way, potential targets for AS were obtained.

### 2.3 Network construction and visualization

#### 2.3.1 Drug-active-constituent–target network construction

The common targets of active constituents of leech and AS were obtained using the intersection of the Venn diagram from the Microbiology Online website (http://www.bioinformatics.com.cn/). The information of common targets of leech and AS was imported into Cytoscapev 3.8.2 [29] to indicate the relationship of potential targets.

#### 2.3.2 Construction of protein–protein interaction (PPI) network

The PPI network was constructed in STRING 11.0 database [30] (https://string-db.org/) with the species set to “*Homo sapiens*” and parameters kept at default settings. The results were imported into Cytoscape 3.8.2 software for topological property analysis to obtain PPI network maps, and the core targets in the PPI network were filtered by degree value ≥ 2 times of the median.

### 2.4 GO and KEGG functional pathway enrichment analyses

By recording the common targets of leech and AS into the Metascape platform [31](http://metascape.org/gp/index.html) and setting P < 0.01, GO functional analysis was performed to characterize the functions of gene targets into cellular, molecular, and biological functions. KEGG enrichment analysis was performed to obtain the signaling pathways enriched by common targets of leech and AS. The results were visualized using the Microbiology Online website.

The leech–AS target–pathway network was constructed using CytoScape 3.8.2, and the network topology parameters of active constituents and targets were analyzed to determine the core targets.

### 2.5 Molecular docking

The Uniprot database [32] (https://www.uniprot.org/) was used to search the protein structures of the core targets in the PPI network and to screen the target proteins that could be used for molecular docking under the following conditions: low resolution, presence of ligand molecules, and single chain structure. The SDF format corresponding to the active constituent was obtained using the Pubchem database (https://pubchem.ncbi.nlm.nih.gov) and imported into OpenBabelGUI2.4.1 to obtain the 3D structure of the active constituent. Finally, the protein–drug molecule docking model was drawn using AutoDockTools1.5.7 and Pymol2.2.0 software.

### 2.6 Construction of mouse model of AS

#### 2.6.1 Animals

Ten 5- to 6-week-old male C57BL/6 mice and 50 male ApoE^−/−^ mice were purchased from Guangdong Pharmachem Biotechnology Co. [license number: SCXK (Guangdong) 2020-0054]. The average body weight of animals was 27 ± 1 g (10–11 weeks old). They were housed in individual ventilated cages in the SPF environment at the First Affiliated Hospital of Guangdong Pharmaceutical University, with free access to water and food and 12 h light/dark cycle. The ambient temperature was maintained at approximately 25 °C. They were acclimatized for 1 week. All animal experiments and operations were approved by the Institutional Animal Care and Use Committee of the First Affiliated Hospital of Guangdong Pharmaceutical University.

#### 2.6.2 Grouping

According to the body weight, the animals were randomly divided into the following groups: model (MOD); blank control (CON); positive control (Simvastatin; SVT); and low, medium, and high doses of proprietary Chinese medicine containing leeches (L-, M − , and H-Leech, respectively). The details of each group are as follows. (1) CON group: included 10C57BL/6 mice feeding on normal chow and equal amount of purified water; (2) MOD group: included 10 ApoE^−/−^ mice feeding on high-fat chow and equal amount of purified water; (3) SVT group: included 10 ApoE^−/−^ mice feeding on high-fat chow; each mouse daily gavaged with 5 mg/kg/day simvastatin at a fixed time; (4) L-, M − , and H-Leech groups: included 10 ApoE^−/−^ mice in each group feeding on high-fat diet; each group were gavaged leech with 0.45, 0.9, and 1.8 mg/kg/day, respectively, once a day at a fixed time.

High-fat feed containing 20% fat and 1.25% cholesterol was purchased from Jiangsu SyiePharma Bioengineering Co., LTD. The feeding period was 8 weeks. After 24 h of fasting and water deprivation, mice were anesthetized. Blood was taken from the eyes. The mice underwent laparotomies along the abdomen's midline to the sternum's angle, and their hearts and aortas were removed. The aortas were dissociated immediately and were immersed in 10% neutral buffered formalin tissue fixative for fixation. The remaining heart tissues were placed in a −80 °C freezer for subsequent testing. All mice were reared and operated in accordance with the regulations related to experimental animal welfare and ethics.

#### 2.6.3 Preparation of proprietary Chinese medicine containing leeches

Proprietary Chinese medicine containing leeches was prepared using MaiXueKang capsules, in which the main raw material is leech. The manufacturing process of MaiXueKangCapsules is to use low temperature control technology and micronization technology to incorporate fresh leeches into medicine, which retains the active ingredients of leeches completely. Clinical studies have demonstrated its effectiveness in the treatment of atherosclerosis [[33], [34], [35]]. The capsules were purchased from Chongqing Dopotai Pharmaceutical Co (Z10970056, China).

#### 2.6.4 Western blotting

Expression levels of SRC were analyzed using western blotting. Myocardial tissue frozen at −80 °C was ground in liquid nitrogen, lysed with RIPA lysis solution, and centrifuged (4 °C,1200 rpm,15min). The supernatant was used to assess the protein concentration using BCA method. The proteins were separated via 10% SDS-PAGE electrophoresis (Beyotime, China) and transferred to a PVDF membrane (Beyotime, China). After blocking the membrane with blocking solution (Beyotime, China) for 80 min at room temperature, the membrane was incubated with SRC antibody (cell signaling, United States) at 4 °C overnight. The next day, the membranes were washed three times with TBST and incubated with HRP-conjugated secondary antibody (cell signaling, United States) for 1 h. After washing the membranes three times with TBST, the immunoreactive bands were visualized via immunoblotting with enhanced chemiluminescent substrate added dropwise to the membranes after exposure.

#### 2.6.5 Detection of lipid deposition and plaque

Mouse aortic samples were snap frozen in a −25 °C freezer for 3–5 min; 8-μm thick sections were cut, fixed in 4% paraformaldehyde, and stained with Oil Red O to visualize aortic lipid deposition and plaque.

### 2.7 Statistical analysis

Statistical analysis was performed using GraphPad Prism software 8.0.2. Among multiple groups were analyzed using a multi-factor analysis of variance (ANOVA) to compare more than two groups. We examined the collected data, which were all normally distributed. P value of less than 0.05 was indicated that the difference was statistically significant.

## 3 Results

### 3.1 Screening of potentially active compounds and action targets of leeches

A total of 59 chemical components of leech were collected from the comprehensive related literature, and 34 active chemical components with good ADME properties were screened using the Swiss ADME database. The basic information of the main active constituents of leech is shown in Table 1.

**Table 1:** Main active ingredients of leech.

Number	Active Ingredients	PubChemID
SZ1	Adenine	PCID190
SZ2	Proline	PCID614
SZ3	Glycine	PCID750
SZ4	Glycerol	PCID753
SZ5	Histamine	PCID774
SZ6	Niacin	PCID938
SZ7	Palmitic acid	PCID985
SZ8	Succinic acid	PCID1110
SZ9	Taurine	PCID1123
SZ10	Uracil	PCID1174
SZ11	Xanthine	PCID1188
SZ12	Alanine	PCID5950
SZ13	Serine	PCID5951
SZ14	Aspartic Acid	PCID5960
SZ15	Lysine	PCID5962
SZ16	Tyrosine	PCID6057
SZ17	Leucine	PCID6106
SZ18	Methionine	PCID6137
SZ19	Phenylalanine	PCID6140
SZ20	Hexanal	PCID6184
SZ21	Histidine	PCID6274
SZ22	Valine	PCID6287
SZ23	Threonine	PCID6288
SZ24	Tryptophan	PCID6305
SZ25	Isoleucine	PCID6306
SZ26	3-Indole-carbaldehyde	PCID10256
SZ27	2-Piperidone	PCID12665
SZ28	Methyl 14-methyl pentadecanoate	PCID21205
SZ29	Methyl 12-methyltetradecanoate	PCID21206
SZ30	Glutamic acid	PCID33032
SZ31	Methyl 11-hexadecenoate	PCID11277200
SZ32	Methyl 4-methyltetradecanoate	PCID25001327
SZ33	Hypoxanthine	PCID135398638
SZ34	Hirudin A	PCID101844805

Overall, 2671 leech-acting genes obtained from 34 active chemical components after screening by Swiss Target Prediction platform, and 402 action targets of leech were obtained by eliminating the targets with probability <10%, and 291 drug action targets were obtained by deleting duplicate terms.

### 3.2 AS-related gene targets

A total of 4688 AS-related genes could be queried in the GeneCards database, and 1172 potential AS-related gene targets were obtained by screening genes with relevance score >1.1164.

### 3.3 Network of targets of active constituents of leech and AS

The 291 drug-action target genes corresponding to the active constituents of leech were intersected with 1172 potential AS-related target genes using the Microbiology Online website, and 89 common targets were obtained (Fig. 1). The “drug-active-constituent–target” visualization network constructed by Cytoscape is shown in Fig. 2, and the information of the components with the top 10° values is shown in Table 2.

**Fig. 1:**
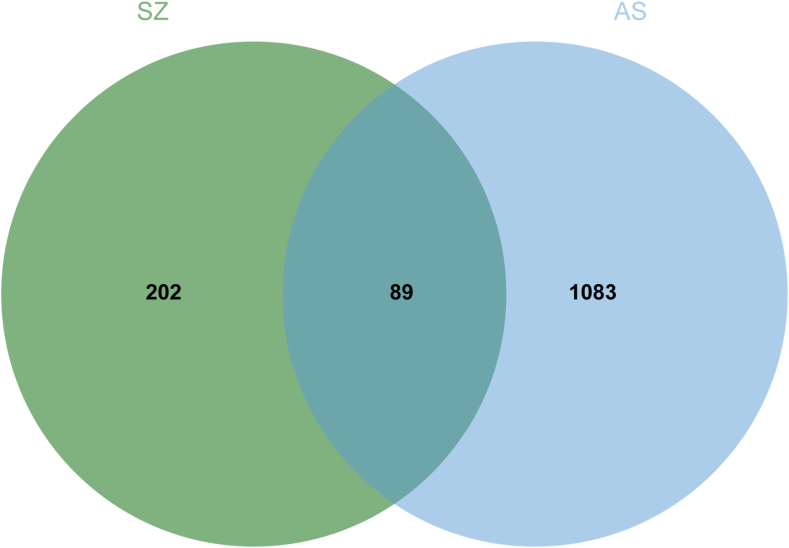
Venn diagram of intersection of targets of active components (drug) and atherosclerosis (AS).

**Fig. 2:**
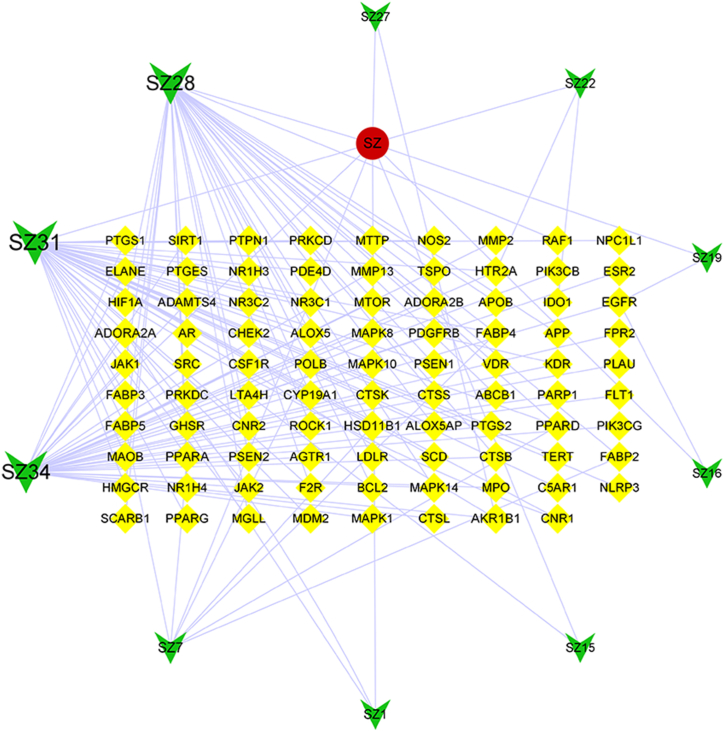
Drug-active-constituent–target network diagram.

**Table 2:** Top 10 component degree values.

Serial number	name	Degree	Intermediary Center Degree	Proximity to center	Neighborhood connectivity
1	SZ31	44	0.4723	0.4901	1.9535
2	SZ34	38	0.4727	0.4670	1.6842
3	SZ28	37	0.3729	0.4626	1.9730
4	SZ7	8	0.0130	0.3640	3.6250
5	SZ22	3	0.0208	0.3511	4.6667
6	SZ15	2	0.0202	0.3486	5.5000
7	SZ1	3	0.0006	0.3486	6.5000
8	SZ27	2	0.0006	0.3486	6.5000
9	SZ19	2	0.0018	0.3486	6.5000
10	SZ16	2	0.0018	0.3486	6.5000

### 3.4 PPI network of targets of active components of leech and AS-related targets

**Fig. 3:**
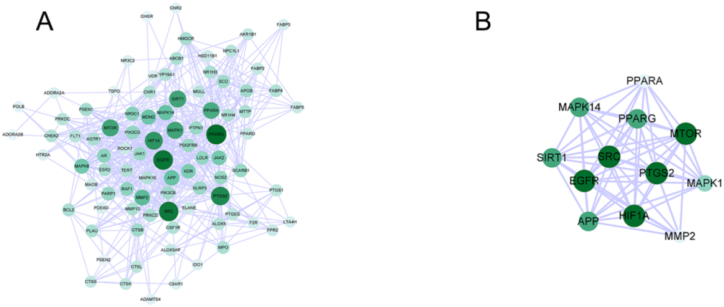
Protein-protein interaction(PPI) network and key targets of leeches in the treatment of AS. The darker the color, the larger the circle, and the larger the degree value, the more critical the position in the target point. (For interpretation of the references to color in this figure legend, the reader is referred to the Web version of this article.)

The 89 common targets were imported into STRING 11.0 database to obtain the interaction relationship network, and the results were saved as text and imported into Cytoscape 3.8.2. In total, 94 nodes and 1240 interaction links were observed in the PPI network (Fig. 3A). The median degree value of the whole network nodes was calculated to be 12. The nodes with degree value ≥ 2 times the median value (degree value ≥ 24) were used as the key targets, and a total of 12 key targets for AS treatment by leech were screened (Fig. 3B). The information of the core targets with degree values in the top-10 positions is shown in Table 3.

**Table 3:** The top 10 key target Degree Values.

Serial number	name	Degree
1	PPARG	46
2	EGFR	44
3	SRC	43
4	PTGS2	39
5	HIF1A	36
6	SIRT1	32
7	MAPK1	32
8	MTOR	32
9	PPARA	30
10	MMP2	26

### 3.5 GO and KEGG pathway enrichment analyses

**Fig. 4:**
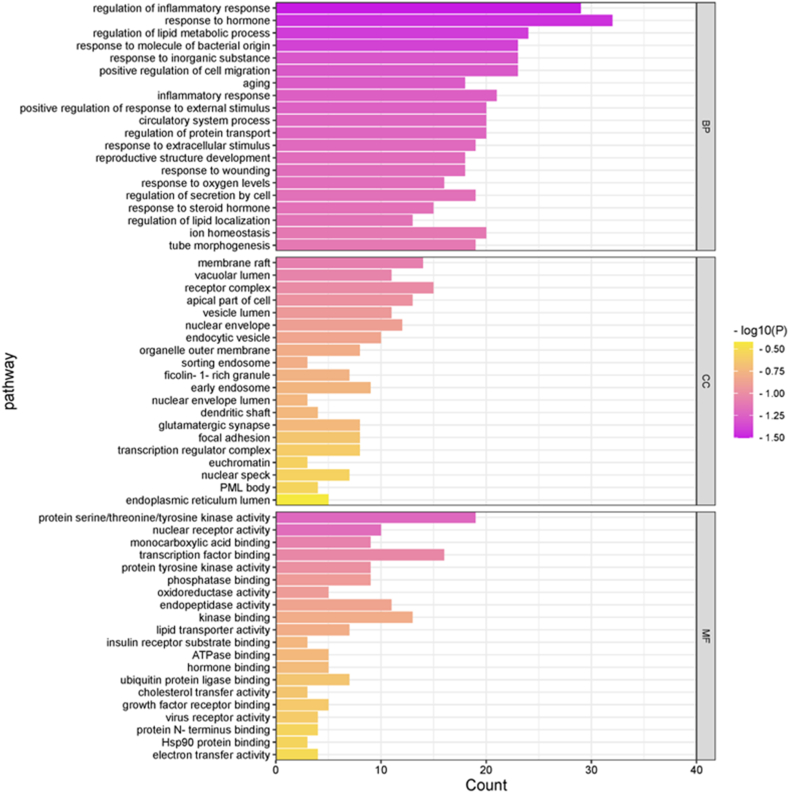
GO pathway enrichment analysis. The darker colors indicat higher significance. (For interpretation of the references to color in this figure legend, the reader is referred to the Web version of this article.)

The results of GO and KEGG pathway enrichment analyses of the common gene targets (according to P < 0.01) using Metascape database revealed 4247, 583, and 89 entries, respectively, related to biological process (BP; including regulation of lipid metabolic process, response to hormone, inflammatory response, membrane raft, and vacuolar lumen), molecular function (MF; including protein serine/threonine/tyrosine kinase activity, phosphatase binding, and tyrosine kinase activity), and cell composition (CC; including membrane raft, vacuolar lumen, and receptor complex). The top-20 enrichment results are shown in the form of bar graphs, The darker colors indicat higher significance (Fig. 4).

**Fig. 5:**
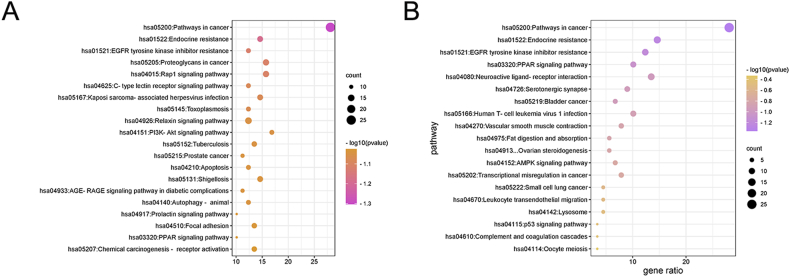
KEGG pathway enrichment analysis. The size of the circles represents the number of genes enriched; darker the color, smaller the P value and higher the significance of enrichment. (For interpretation of the references to color in this figure legend, the reader is referred to the Web version of this article.)

We obtained 221 pathways by KEGG pathway enrichment analysis (P < 0.01) based on relevant literature. The top-20 pathways were selected to obtain a visual enrichment bubble map. The analysis revealed that the pathways that may play a role were mainly cancer pathway, EGFR tyrosine kinase inhibitor resistance, PPAR signaling pathway, Rap1 signaling pathway, PI3K-Akt signaling pathway, etc. (Fig. 5A,B and Table 4).

**Table 4:** Pathway-target information.

Access	LogP	count	Target Points
hsa05200:Pathways in cancer	−22.6498	25	AGTR1,AR,BCL2,CSF1R,EGFR,ESR2,F2R,MTOR,HIF1A,JAK1,JAK2,MDM2,MMP2,NOS2,PDGFRB,PIK3CB,PPARD, PPARG,MAPK1,MAPK8,MAPK10,PTGS2,RAF1, ROCK1,TERT
hsa01522:Endocrine resistance	−17.6549	13	BCL2,MAPK14,EGFR,ESR2,MTOR,MDM2,MMP2,PIK3CB,MAPK1,MAPK8,MAPK10,RAF1,SRC,ADORA2A,ADORA2B,CNR1,CSF1R,F2R,FLT1,KDR,PDJFRB, PRKCD,PTGS2 NLRP3,HIF1A,JAK1,JAK2,PIK3CG,ALOX5,LDLR,NOS2,MMP13,CTSS,VDR,PARP1,CTSB, CTSK,CTSL, AGTR1,ROCK1,APOB, PPARG,PSEN1,PSEN2,ALOX5AP,GHSR, PDE4D,PPARA, PTPN1,SIRT1,NR1H3,C5AR1,APP,HTR2A,SCD,MGLL, MAOB,PPARD
hsa01521:EGFR tyrosine kinase inhibitor resistance	−15.2314	11	BCL2,EGFR, MTOR,JAK1,JAK2,KDR,PDGFRB,PIK3CB,MAPK1,RAF1,SRC,MAPK14,CTSL,HIF1A,MDM2,MMP2,PLAU, ROCK1,CSF1R,F2R,FLT1,PIK3CG,AR,ESR2, PPARA,VDR,ABCB1,PTGS2,SIRT1,MAPK8,MAPK10,C5AR1,ELANE,FPR2,MPO,PRKCD, AGTR1,SCARB1,LDLR,NR1H3,PPARD,NOS2,PTGS1,PSEN1,TERT, CHEK2, NLRP3,NR3C2
hsa05205:Proteoglycans in cancer	−14.8638	14	MAPK14,CTSL, EGFR,MTOR,HIF1A,KDR,MDM2,MMP2,PIK3CB,PLAU, MAPK1,RAF1,ROCK1,SRC
hsa04015:Rap1 signaling pathway	−14.7176	14	ADORA2A,ADORA2B,CNR1,MAPK14,CSF1R,EGFR,F2R,FLT1,KDR,PDGFRB,PIK3CB,MAPK1,RAF1,SRC
hsa04625:C-type lectin receptor signaling pathway	−13.8647	11	MAPK14,MDM2,PIK3CB,PRKCD, MAPK1,MAPK8,MAPK10,PTGS2,RAF1,SRC,NLRP3
hsa05167:Kaposi sarcoma-associated herpesvirus infection	−13.7143	13	MAPK14,MTOR,HIF1A,JAK1,JAK2,PIK3CB,PIK3CG,MAPK1,MAPK8,MAPK10,PTGS2,RAF1,SRC
hsa05145:Toxoplasmosis	−13.5014	11	ALOX5,BCL2,MAPK14,JAK1,JAK2,LDLR,NOS2,PIK3CG,MAPK1,MAPK8,MAPK10
hsa04151:PI3K-Akt signaling pathway	−12.8548	15	BCL2,CSF1R,EGFR,F2R,FLT1,MTOR,JAK1,JAK2,KDR,MDM2,PDGFRB,PIK3CB,PIK3CG,MAPK1,RAF1
hsa04926:Relaxin signaling pathway	−12.8140	11	MAPK14,EGFR,MMP2,MMP13,NOS2,PIK3CB,MAPK1,MAPK8,MAPK10,RAF1,SRC
hsa05152:Tuberculosis	−12.6572	12	BCL2,MAPK14,CTSS,JAK1,JAK2,NOS2,MAPK1,MAPK8,MAPK10,RAF1,SRC,VDR
hsa04210:Apoptosis	−12.5587	11	PARP1,BCL2,CTSB, CTSK,CTSL, CTSS,PIK3CB,MAPK1,MAPK8,MAPK10,RAF1
hsa05215:Prostate cancer	−12.5254	10	AR,BCL2,EGFR, MTOR,MDM2,PDGFRB,PIK3CB,PLAU, MAPK1,RAF1
hsa04933:AGE-RAGE signaling pathway in diabetic complications	−12.3897	10	AGTR1,BCL2,MAPK14,JAK2,MMP2,PIK3CB,PRKCD, MAPK1,MAPK8,MAPK10
hsa04140:Autophagy - animal	−12.3848	11	BCL2,CTSB, CTSL,MTOR,HIF1A,PIK3CB,PRKCD, MAPK1,MAPK8,MAPK10,RAF1
hsa05131:Shigellosis	−12.3658	13	BCL2,MAPK14,EGFR, MTOR,MDM2,PIK3CB,PRKCD, MAPK1,MAPK8,MAPK10,ROCK1,SRC,NLRP3
hsa04917:Prolactin signaling pathway	−12.2081	9	MAPK14,ESR2,JAK2,PIK3CB,MAPK1,MAPK8,MAPK10,RAF1,SRC
hsa04510:Focal adhesion	−12.0864	12	BCL2,EGFR,FLT1,KDR,PDGFRB,PIK3CB,MAPK1,MAPK8,MAPK10,RAF1,ROCK1,SRC
hsa03320:PPAR signaling pathway	−11.9275	9	FABP4,FABP2,FABP3,FABP5,PPARA, PPARD,PPARG,SCD,NR1H3
hsa05207:Chemical carcinogenesis - receptor activation	−11.8123	12	AR,BCL2,EGFR,ESR2,MTOR,JAK2,PIK3CB,PPARA, MAPK1,RAF1,SRC,VDR

### 3.6 Construction of leech-AS-target-pathway network diagram

**Fig. 6:**
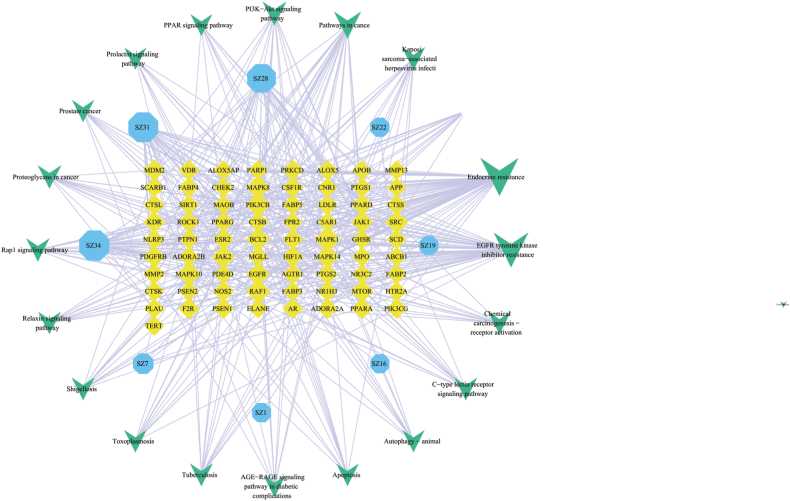
Active-component–AS target–pathway network.

The results of GO and KEGG pathway enrichment analyses were combined to screen potential pathways of leech for the treatment of AS that corresponded to the active constituent and target one by one. The relevant data were recorded into Cytoscape 3.8.2 to construct the active constituent–target–pathway network (Fig. 6). The network diagram contained 102 nodes and 432 edges. MAPK1 was predicted to be the most important target of leech for AS treatment, whereas PIK3CB, RAF1, MAPK10, MAPK8, MAPK14, EGFR, BCL2, SRC, and JAK2 were also important targets according to the network analysis (Table 5).

**Table 5:** Top 10 AS targets.

Target Points	Degree	Intermediary Center Degree	Proximity to center	Neighborhood connectivity
MAPK1	20	0.0291	0.5102	17.6000
PIK3CB	18	0.0239	0.5000	18.2778
RAF1	16	0.0233	0.5051	19.0000
MAPK10	15	0.0173	0.4854	19.1333
MAPK8	15	0.0173	0.4854	19.1333
MAPK14	14	0.0201	0.4950	20.4286
EGFR	14	0.0508	0.4808	19.0714
BCL2	14	0.0191	0.4950	19.6429
SRC	13	0.0130	0.4762	20.0000
JAK2	12	0.0161	0.4854	23.3333

### 3.7 Molecular docking

**Fig. 7:**
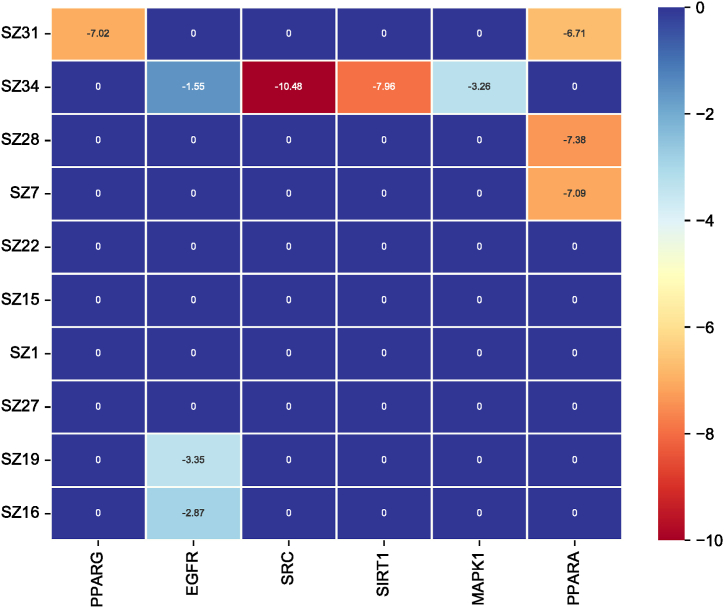
Binding energy diagram. The 0 represents no interaction between the active constituent and target, and binding energy < −10 kcal⸱mol^−1^ represents strong binding ability and < −5.0 kcal⸱mol^−1^ represents good binding ability.

**Fig. 8:**
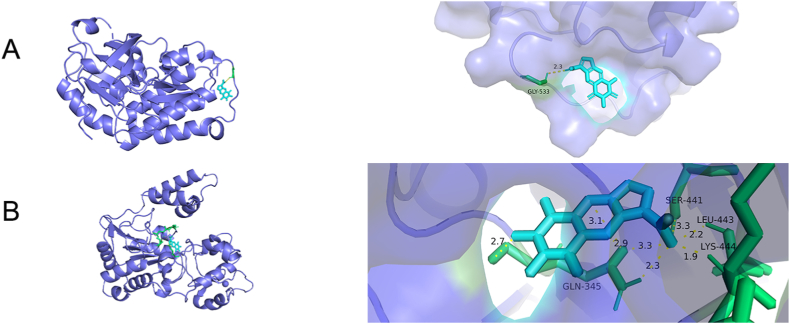
Molecular docking A Hirudin A-protein tyrosine kinase, B Hirudin A-nicotinamide adenosine dinucleotide (NAD)-dependent deacetylase.

The top-10 active constituents (Table 2) and core targets (Table 3; PTGS2, HIF1A, MTOR, and MMP2 were not eligible for molecular docking, so they were excluded) were obtained from literature review and this study, and molecular docking was performed according to the existence of interaction between active constituents and core targets. The binding energies of the docking results are shown in Fig. 7. The binding energy < −5.0 kcal⸱mol^−1^ was selected for the demonstration of molecular docking results. Molecular docking results for Hirudin A-protein tyrosine kinase and Hirudin A-nicotinamide adenosine dinucleotide (NAD)-dependent deacetylase are shown in Fig. 8A and B.

### 3.8 Effect of atherosclerotic plaques in a mouse model of AS

**Fig. 9:**
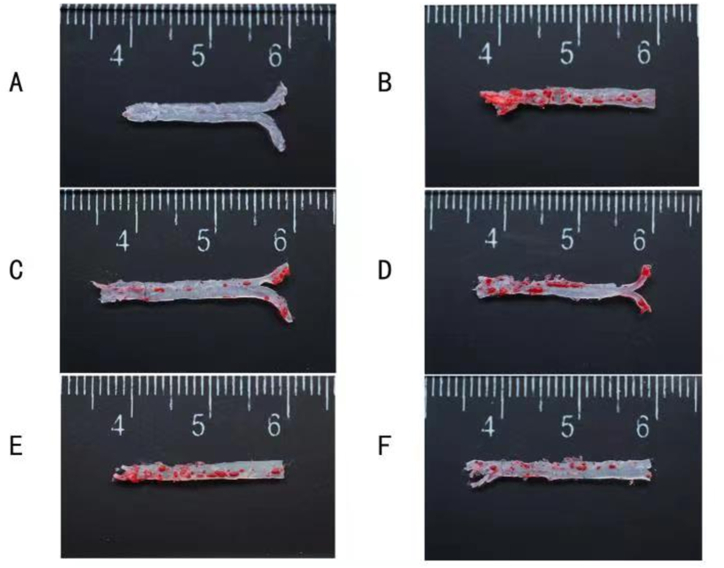
Results of oil red O staining in the atherogenic zone of ApoE^−/−^ mice. A: blank control group (CON); B: model group (MOD); C: simvastatin group (STV); D: low-dose gavage group (L-Leech); E: medium-dose gavage group (M-Leech), and F: high-dose gavage group (H-Leech). (For interpretation of the references to color in this figure legend, the reader is referred to the Web version of this article.)

**Fig. 10:**
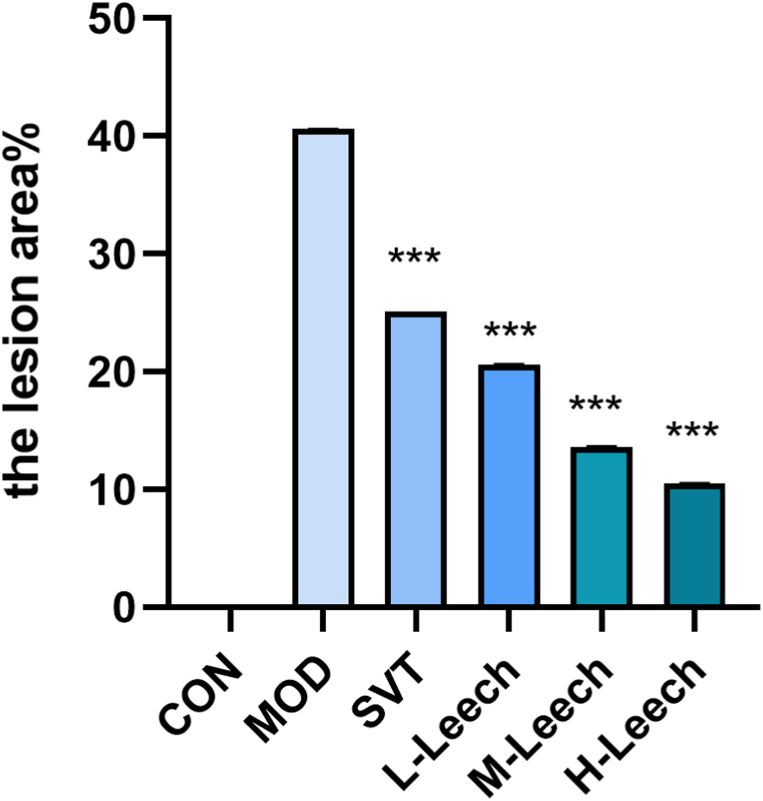
The proportion of the lesion area by oil red O staining, n = 4. (For interpretation of the references to color in this figure legend, the reader is referred to the Web version of this article.)

**Fig. 11:**
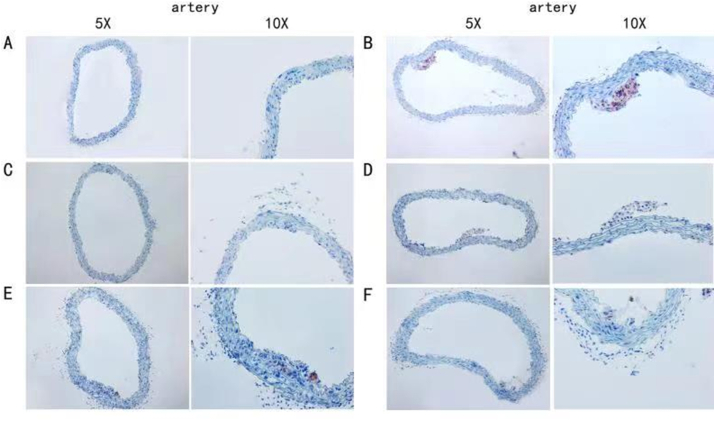
Results of oil red O staining in atherogenic areas of ApoE^−/−^ mice. A: blank control group (CON); B: model group (MOD); C: simvastatin group (STV); D: low-dose gavage group (L- Leech); E: medium-dose gavage group (M-Leech), and F: high-dose gavage group (H-Leech). (For interpretation of the references to color in this figure legend, the reader is referred to the Web version of this article.)

**Fig. 12:**
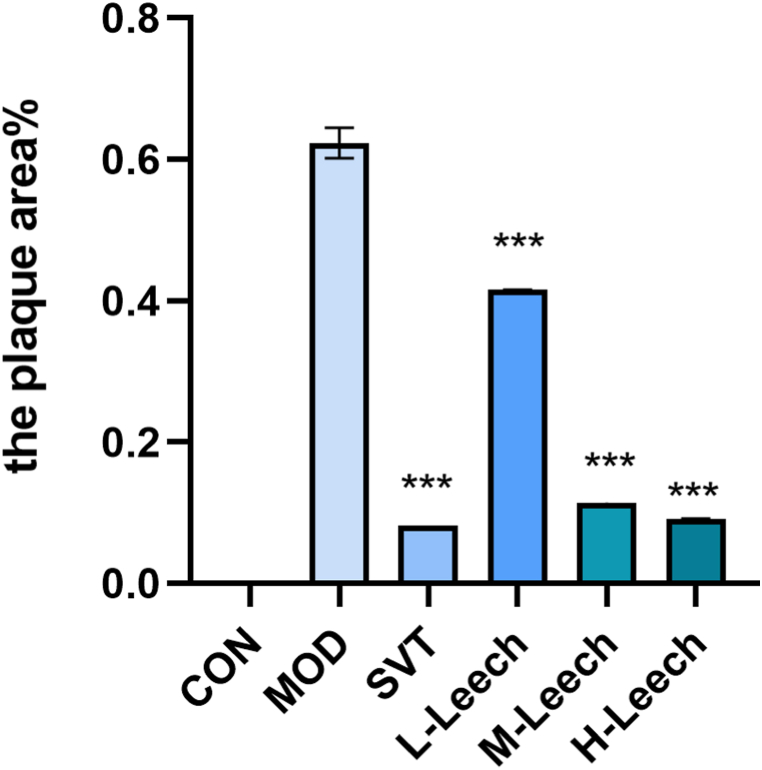
The proportion of the atherogenic areas by oil red O staining, n = 4. (For interpretation of the references to color in this figure legend, the reader is referred to the Web version of this article.)

Oil red O staining revealed a smooth and clean aorta in CON group and the most plaque in MOD group. STV and L-, M − , and H-Leech groups exhibited less plaque than MOD group, and L-, M − , and H-Leech groups exhibited less plaque than STV group. Fig. 9(A-F)and Fig. 11(A-F)are the results of oil red O staining in the atherogenic zone of ApoE^−/−^ mice. nd their quantification results are shown in Fig. 10, Fig. 12.

### 3.9 Western blotting

**Fig. 13:**
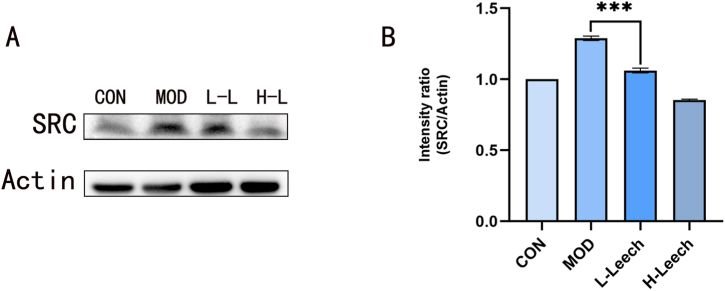
Protein expression levels of SRC were measured using Western blot; The quantitative results of SRC were analyzed using ImageJ, n = 3. Values are expressed as the mean ± SD (***P < 0.05).

Western blotting was performed to assess the expression of SRC and their phosphorylation levels, which revealed the medicinal efficacy of leech. One of the most significant signaling target involved in the prevention of AS is SRC. We previously demonstrated that SRC is involved in the activation of MAPK signaling pathway, promoting cell inflammatory response and proliferation migration [19,36]. SRC is a potential therapeutic molecule for reducing morbidity and mortality related to cardiovascular diseases. We performed western blotting to assess the expression of SRC in the myocardial tissue of mice. The expression of SRC was the least in H-Leech group and significantly higher in MOD and L- and M-Leech groups (P < 0.05; Fig. 13A),and their quantification results are shown in Fig. 13B. These findings suggested that leech prevents AS by modulating SRC.

## 4 Discussion

The pathogenesis of AS is very complex, and lipid metabolism disorder is considered to be one of the main causes. Lipid metabolism involves the regulation of multiple targets and pathways, and the treatment drugs are relatively single. However, no previous studies have reported satisfactory results in mechanistic studies of leeches on AS. Therefore, it is necessary to find alternative or complementary therapies for AS. Different from other mechanistic studies, we used both virtual method and in vitro experiments for validation. Here, we first identified the active component of leeches associated with AS through network pharmacology, and we demonstrated that leeches reduced AS plaque size by modulating SRC protein targets. The results suggest that leeches reduce the expression of SRC by counterregulating the related signaling pathways to slow the progression of AS.

In this study, network pharmacological methods were used to analyze the active constituents of leech against AS. The results revealed that hirudin A, palmitic acid, and adenine were the main active constituents. Hirudin is a well-studied compound, which has anticoagulant, antithrombotic, and anti-inflammatory effects. However, some studies have reported that hirudin can activate AS inflammatory factors in the process of response to endothelial injury, thus accelerating the formation of lesions in AS [13,37,38]. Palmitic acid is the most abundant saturated higher fatty acid in dosing plasma lipids. Studies have reported that palmitic-acid-treated human arterial endothelial cells have significantly higher expression of the proinflammatory cytokine IL-6, which is involved in the inflammatory response inducing AS development [39,40].

PPI network revealed that the active components in leech may act through PPARG, EGFR, SRC, PTGS2, HIF1A, SIRT1, MAPK1, and other common targets. PPARG is involved in multitarget and multipathway anti-AS actions such as inflammatory response, myocardial vascular smooth muscle value-added, and vascular endothelial function. The expression of TNF-α, IL-1β, and IL-6 in rat aorta was significantly increased after intervention with PPARG inhibitor in a high-fat-diet-induced rat model, indicating that PPARG has a clear anti-inflammatory effect [[41], [42], [43]]. SRC is involved in the progression of AS by promoting cell proliferation, migration, and lipid accumulation. SRC is involved in the activation of MAPK signaling pathway, promoting cell inflammatory response and proliferation migration [19,36]. EGFR can activate multiple signaling pathways, and active EGFR can promote vascular smooth muscle cell proliferation through PI3K/Akt, E-Cadherin/β-Catenin/TcF, and other signaling pathways [44,45].

The results of KEGG pathway enrichment analysis revealed that the potential mechanism of action of leech in AS is mainly related to the regulatory pathways involving cancer pathway, EGFR tyrosine kinase inhibitor resistance, PPAR signaling pathway, Rap1 signaling pathway, and PI3K-Akt signaling pathway. The PPAR signaling pathway regulates endogenous PPAR during the regulation of lipid metabolism, inhibits the expression of HMG-CoA, and exerts anti-inflammatory and anti-AS effects [15,[46], [47], [48]]. The PI3K-Akt signaling pathway regulates downstream mTOR proteins, and selective inhibition of the PI3K/Akt/mTOR signaling pathway can inhibit the inflammatory response, thereby stabilizing AS-prone plaques. PI3K/Akt signaling pathway is strongly involved in the occurrence of AS via its role in vascular smooth muscle proliferation and inflammatory cellular responses, which contribute to the development and progression of AS [49,50].

The molecular docking results revealed that hirudin A exhibited the strongest binding energy of −10.48 kcal⸱mol^−1^ to protein tyrosine kinase. SRC protein kinase will be further investigated as a new therapeutic target for drugs that specifically antagonize SRC protein kinase activity [36,[51], [52], [53], [54], [55]]. Activating blood herbs are involved in the inflammatory response, neuroprotection, and angiogenesis through PI3K-Akt signaling pathway, and the key targets include SRC, which is consistent with the dialectical treatment of bruising pathology in traditional Chinese medicine [56,57]. The above pathways and targets are involved in lipid metabolism, AS inflammatory factors, vascular smooth muscle cell proliferation, and other processes, which contribute to the prevention of AS in multiple ways. Our findings suggested that leech makes the plaque smaller and has an ameliorative and preventive effect on AS.

Our analysis indicated that SRC may be a promising target that is closely linked to AS. This should be further investigated. Therefore, after determining the effect of leeches on lowering lipid levels and stabilizing plaques, we measured the level of SRC in the myocardial tissue. The SRC levels were lower in L-, M − , and H-Leech groups than in MOD group. The study suggested that leeches attenuate the progression of AS by reverse regulating the related signaling pathways to lower the expression of SRC. However, there is no direct evidence regarding the mechanism by which leech accurately regulates the SRC pathway. In future studies, we hope to perform validation of the entire pathway and co-immunoprecipitation experiments to explore the direct mechanism.

## 5 Conclusion

Research on Chinese herbal medicine mainly focuses on activating blood and dissolving statis, dreging the meridims and phlegm but ignores its multicomponent, multitarget, and multipathway action characteristics. Both should be combined to improve the precise use of leech as a single drug.

In this study, the main active compounds and pharmacological effects of leech were initially investigated by applying network pharmacology. The results confirmed that several active constituents of leech can act on different targets proteins, and each target is involved in different biological processes, molecular functions, and signaling pathways. The experiment verified the SRC as key targets have a regulating effect on atherosclerosis. This provided a theoretical basis for the study of leech at the molecular biology level and a direction for the subsequent animal and cellular studies.

However, due to the limitations of network pharmacology, this study could not consider the drug composition, complex effects that occur during decoction formation, and metabolic processes of the drug in vivo [56]. The present study is mainly based on the results of network pharmacology and data analysis. Based on this, further animal or cellular screening will be conducted for experimental validation, and the main regulatory targets of the active components of leech and regulation of AS through microRNA pathway will be clarified.

## Data availability

Data will be made available on request.

## CRediT authorship contribution statement

**Qing Lv:** Writing – review & editing, Writing – original draft, Visualization, Validation, Supervision, Software, Resources, Methodology, Investigation, Data curation. **Mengyi Li:** Writing – review & editing, Writing – original draft, Visualization, Validation, Supervision, Software, Resources, Methodology, Investigation, Data curation. **Ziyun Wen:** Writing – review & editing. **Qianqian Han:** Methodology, Conceptualization. **Liang Wei:** Writing – review & editing. **Jisheng Chen:** Validation, Supervision, Resources, Project administration, Investigation, Funding acquisition, Formal analysis, Conceptualization. **Yunyun Pan:** Supervision, Resources, Project administration, Investigation, Funding acquisition.

## Declaration of competing interest

The authors declare that they have no known competing financial interests or personal relationships that could have appeared to influence the work reported in this paper.

## Figures and Tables

**figs1:**
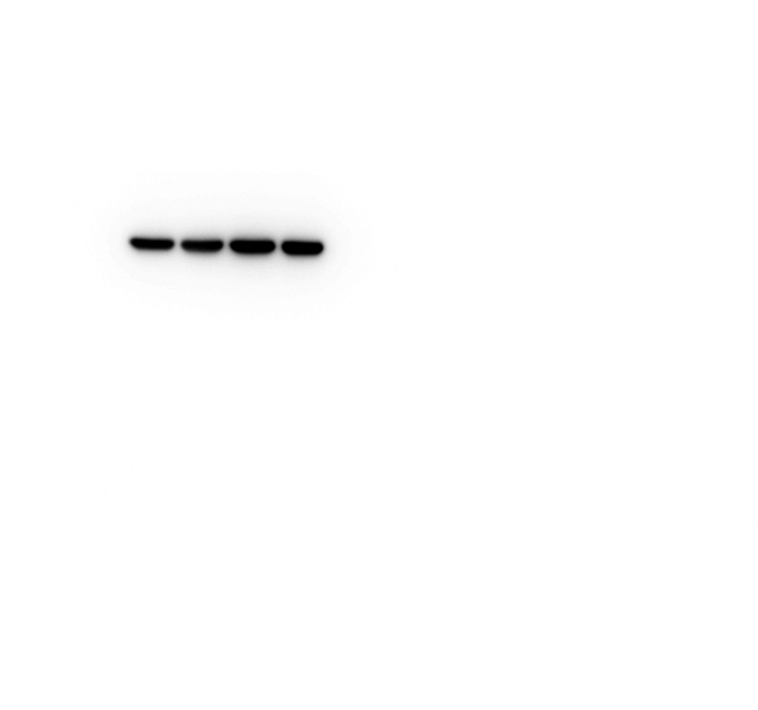


**figs2:**
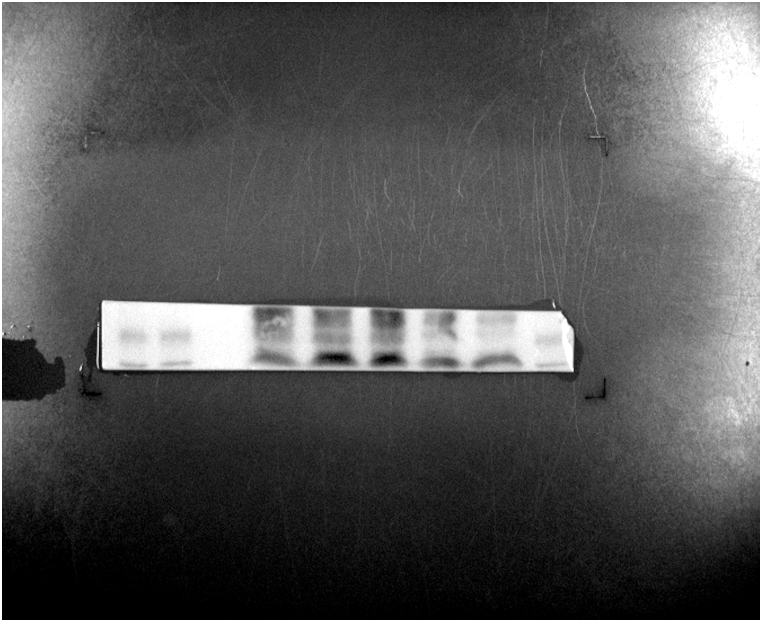


## Abbreviations

AS: atherosclerosis
SRC: sarcoma
MAPK: mitogen-activated protein kinase
EGFR: epidermal growth factor receptor
PIK3CB: Recombinant Phosphoinositide-3-Kinase Catalytic Beta Polypeptide
PPAR: Peroxisome proliferator-activated receptor
PI3K: phosphatidylinositol 3 kinase
Akt: protein kinase B
AGE: advanced glycation end products
RAGE: receptor for advanced glycation end products
SMILES: Simplified molecular input line entry system
PPI: protein–protein interaction
GO: gene ontology
KEGG: Kyoto Encyclopedia of Genes and Genomes
MOD: Module
CON: Control
SVT: Simvastatin
RIPA: Radio-Immunoprecipitation Assay
SDS: Sodium dodecyl sulfate
PAGE: Polyacrylamide gel electrophoresis.
TBST: TBS with Tween-20.
BCA: bicinchoninicacid
